# Modified recurrent equation-based cubic spline interpolation for missing data recovery in phasor measurement unit (PMU)

**DOI:** 10.12688/f1000research.73182.3

**Published:** 2023-12-18

**Authors:** Shruthi Thangaraj, Vik Tor Goh, Timothy Tzen Vun Yap

**Affiliations:** 1 Faculty of Engineering, Multimedia University, Cyberjaya, Selangor, 63100, Malaysia; 2 Faculty of Computing and Informatics, Multimedia University, Cyberjaya, Selangor, 63100, Malaysia

**Keywords:** phasor measurement unit, missing data, data recovery, smart grid, interpolation, cubic spline, data quality, data pre-processing

## Abstract

**Background:**

Smart grid systems require high-quality Phasor Measurement Unit (PMU) data for proper operation, control, and decision-making. Missing PMU data may lead to improper actions or even blackouts. While the conventional cubic interpolation methods based on the solution of a set of linear equations to solve for the cubic spline coefficients have been applied by many researchers for interpolation of missing data, the computational complexity increases non-linearly with increasing data size.

**Methods:**

In this work, a modified recurrent equation-based cubic spline interpolation procedure for recovering missing PMU data is proposed. The recurrent equation-based method makes the computations of spline constants simpler. Using PMU data from the State Load Despatch Center (SLDC) in Madhya Pradesh, India, a comparison of the root mean square error (RMSE) values and time of calculation (ToC) is calculated for both methods.

**Results:**

The modified recurrent relation method could retrieve missing values 10 times faster when compared to the conventional cubic interpolation method based on the solution of a set of linear equations. The RMSE values have shown the proposed method is effective even for special cases of missing values (edges, continuous missing values).

**Conclusions:**

The proposed method can retrieve any number of missing values at any location using observed data with a minimal number of calculations.

## Introduction

The worldwide growing power systems highlight the need for better monitoring and control mechanisms to avoid major blackouts. Smart grids are intelligent systems that facilitate the development of communication, network, and computing technologies, protocols, and standards to integrate power system elements for two-way communication. This time-synchronized high-precision measurement device that is also known as a synchrophasor or Phasor Measurement Unit (PMU), gives clear information on the working of the entire grid. The PMU is used to monitor and control the power grid. It can help in providing real-time measurements by eliminating adverse conditions like blackouts. These combined characteristics of data availability, timeliness, and communication network contribute to the better performance of the PMU system. Although the role, impact, ^1^ architecture, technology, ^2^ applications, functionality, standards, and evolution of PMU (timing, measurement, communication, and data storage) have been released since 1995, the North American Synchro Phasor Initiative (NASPI) has highlighted the importance of data quality. ^3^ Data quality issues, their potential causes, and consequences are elaborated. ^4^^–^^6^ Generally, incomplete or missing data might affect the functionality of the entire system. ^7^ Hence, a way to handle missing values in PMU is mandatory for the effective functioning of the entire grid system.

With the advent of PMU systems, large datasets are generated and finding missing values using traditional cubic interpolation methods take larger computational time with the increase in data size. In this paper, a modified recurrent equation-based method termed the Alpha Method (AM) for PMU missing data problem is proposed. In this approach, a series of linear equations are solved using the modified recurrent equation to obtain a relationship between points on a spline, which is then used to estimate any missing values on the spline. We compare the proposed method to the more traditional method of solving linear equations, namely using tri-diagonal matrix or termed as the Linear Equations Method (LEM) in this paper. The proposed AM is computationally more efficient and takes less time to process than the LEM. Moreover, in real-time systems when the dataset grows progressively, we show that AM is better than LEM.

## Literature review

The need to recover missing values in PMU data is vital to the proper operation of smart grids and the energy infrastructure. Literatures ^5^^–^^7^ indicate that missing data in PMU systems can negatively affect the accuracy of decision-making process and additionally, introduce security risks to the infrastructure. To address this problem, missing values have to be recovered and one of the more popular approaches is utilizing matrix completion. ^8^^–^^12^ Despite that, this approach is still largely theoretical and even so, viable methods utilizing this approach have only been tested on simulated data.

Alternatively, interpolation-based missing data recovery techniques ^13^^–^^15^ propose the reconstruction of missing values by a spatial interpolation or spatio-temporal interpolation of the values. Some work ^16^^,^^17^ even suggested advanced approaches utilizing *k*-nearest neighbors and recurrent relation-based interpolations. However, in interpolation-based techniques, historical data such as channel or time data is needed for more accurate calculations. interpolation. As such, there is a need to design effective data recovery methods to work without the need for historical data processing. ^3^ So, a data-driven recovery technique capable of recovering missing entries with available or observed data is much needed. Moreover, the technique should not become overly complex or require high computational time as the size of the data grows.

## Methods

Cubic spline interpolation is a widely used polynomial interpolation method for functions of one variable. Let f be a function from RtoR . It is assumed that the value of f is known only at $x_{1}\leq x_{2.}\leq x_{i}\ldots \leq x_{n}\;\text{and let}\;f\left(x_{i}\right)=a_{i}.$ Piecewise cubic spline interpolation is the problem of finding the $b_{i}$ , $c_{i}$ and $d_{i}$ coefficients of the cubic polynomials ${\mathrm{SF}}_{i}\;\text{for}\;0\leq i\leq n-1$ written in the form:

$${\mathrm{SF}}_{i}\left(x\right)=a_{i}+b_{i}\left(x-x_{i}\right)+c_{i}{\left(x-x_{i}\right)}^{2}+d_{i}{\left(x-x_{i}\right)}^{3}$$ (1)

Where x can take any value between $x_{i}$ and $x_{i+1}$ . That is,

$$\;{\mathrm{SF}}_{i}\left(x_{i}\right)=a_{i}$$ (1a)

Let the first-order derivative of equation (1) be:

$${\mathrm{SF}}_{i}^{\prime }\left(x\right)=b_{i}+2c_{i}\left(x-x_{i}\right)+3d_{i}{\left(x-x_{i}\right)}^{2}$$ (2)

The first-order derivative at $x_{i}$ for values of 1≤i≤n−1 will be

$$\;{\mathrm{SF}}_{i}^{\prime }\left(x_{i}\right)=b_{i}$$ (2a)

And the second-order derivative be:

$${\mathrm{SF}}_{i}^{\prime \prime }\left(x\right)=2c_{i}+6d_{i}\left(x-x_{i}\right)$$ (3)

The second-order derivative at $x_{i}$ for values of 1≤i≤n−1 will be:

$${\mathrm{SF}}_{i}^{\prime \prime }\left(x_{i}\right)=2c_{i}$$ (3a)

For a smooth fit between the adjacent pieces, the cubic spline interpolation requires that the following conditions hold:

1. The cubic functions should intersect at the points left and right, for i=0ton−1
$${\mathrm{SF}}_{i}\left(x_{i+1}\right)={\mathrm{SF}}_{i+1}\left(x_{i}\right)=a_{i+1}$$ (4)
2. For each cubic function to join smoothly with its neighbors, the splines should have continuous first and second derivatives at the data points i=1,…,n−1:
$${\mathrm{SF}}_{i}^{\prime }\left(x_{i+1}\right)={\mathrm{SF}}_{i+1}^{\prime }\left(x_{i}\right)=b_{i+1}$$ (5)
$${\mathrm{SF}}_{i}^{\prime \prime }\left(x_{i+1}\right)={\mathrm{SF}}_{i+1}^{\prime \prime }\left(x_{i}\right)=2.c_{i+1}$$ (6)

If $h_{i}$ = $x_{i+1}-x_{i}$ and if $h_{i}$ is equal for all i values, following Revesz, ^17^ the relation between coefficients $a_{i}$ and $c_{i}$ can be resolved:

$$c_{i-1}+4\;c_{i}+c_{i+1}=\frac{3}{h^{2}}\left(a_{i-1}-2a_{i}+a_{i+1}\right)$$ (7)

$$b_{i}=\left(a_{i+1}-a_{i}\right)\frac{1}{h_{i}}-\frac{2c_{i}+c_{i+1}}{3}h_{i}$$ (8)

$$d_{i}=\frac{1}{3.h_{i}}\left(c_{i+1}-c_{i}\right)$$ (9)

Equation (7) represents a system of linear equations for the unknowns $c_{i}$ for 0≤i≤n . As the values of $a_{i}$ are known, the value of $c_{i}$ can be found by solving the tri-diagonal matrix-vector equation Ax=B . While there are *n*+1 numbers of $c_{i}$ constants, equation (7) yields only ( *n*-2) equations. Based on the nature or type of spline assumed two more equations representing the boundary conditions of the spline. In general, two types of splines may be considered: natural cubic spline and clamped cubic spline.

For natural cubic spline interpolation, the following boundary conditions are assumed: $c_{0}=c_{n}=0.0$ . That is, the second derivatives of the splines at the endpoints are assumed to be zero. Based on equation (7), a system of ( *N*+1) linear equations of ( *N*+1) variables can be formulated as:

$$A=\left[\begin{array}{lllllllll} 1 & 0 & 0 & 0 & \cdots & 0 & 0 & 0 & 0\\ 1 & 4 & 1 & 0 & \cdots & 0 & 0 & 0 & 0\\ 0 & 1 & 4 & 1 & \cdots & 0 & 0 & 0 & 0\\ \vdots & \vdots & \vdots & \vdots & \vdots & \vdots & \vdots & \vdots & \vdots \\ 0 & 0 & 0 & 0 & \cdots & 1 & 4 & 1 & 0\\ 0 & 0 & 0 & 0 & \cdots & 0 & 1 & 4 & 1\\ 0 & 0 & 0 & 0 & \cdots & 0 & 0 & 0 & 1 \end{array}\right],\;x=\left[\begin{array}{c} \begin{array}{c} c_{0}\\ c_{1}\\ \vdots \\ c_{n} \end{array} \end{array}\right],\text{and}\;B=\;\left[\begin{array}{c} \begin{array}{c} 0\\ \frac{3}{h^{2}}\left(a_{0}-2a_{1}+a_{2}\right) \end{array}\\ \begin{array}{c} \vdots \\ \frac{3}{h^{2}}\left(a_{n-2}-2a_{n-1}+a_{n}\right)\\ 0 \end{array} \end{array}\right]$$ (10)

For clamped cubic spline interpolation the following boundary conditions are assumed: $b_{0}=f^{\prime }(x_{0}$ ) and $b_{n}=f^{\prime }(x_{n}$ ), where the derivatives $f^{\prime }(x_{0}$ ) and $f^{\prime }(x_{n}$ ), are known constants. Thus, based on the boundary conditions assumed both natural and cubic splines result in *n*+1 system of linear equations. The resulting system of *n*+1 linear equations can be used to get unique solutions by any of the standard methods for solving a system of linear equations.

Once the values of $c_{i}$ are found, the *b*_*i*_ and *d*_*i*_ values can be obtained using equations (8) and (9) respectively. Similarly, under clamped spline interpolation,

$$A=\left[\begin{array}{lllllllll} 2 & 1 & 0 & 0 & \cdots & 0 & 0 & 0 & 0\\ 1 & 4 & 1 & 0 & \cdots & 0 & 0 & 0 & 0\\ 0 & 1 & 4 & 1 & \cdots & 0 & 0 & 0 & 0\\ \vdots & \vdots & \vdots & \vdots & \vdots & \vdots & \vdots & \vdots & \vdots \\ 0 & 0 & 0 & 0 & \cdots & 1 & 4 & 1 & 0\\ 0 & 0 & 0 & 0 & \cdots & 0 & 1 & 4 & 1\\ 0 & 0 & 0 & 0 & \cdots & 0 & 0 & 1 & 2 \end{array}\right],\;x=\left[\begin{array}{c} \begin{array}{c} c_{0}\\ c_{1}\\ \vdots \\ c_{n} \end{array} \end{array}\right]\;\text{and}\;B=\left[\begin{array}{c} \begin{array}{c} \frac{3}{h^{2}}\left(a_{1}-a_{0}\right)-\frac{3}{h}f^{\prime }\left(x_{0}\right)\\ \frac{3}{h^{2}}\left(a_{0}-2a_{1}+a_{2}\right) \end{array}\\ \begin{array}{c} \vdots \\ \frac{3}{h^{2}}\left(a_{n-2}-2a_{n-1}+a_{n}\right)\\ \frac{3}{h}f^{\prime }\left(x_{0}\right)-\frac{3}{h^{2}}\left(a_{n}-a_{n-1}\right) \end{array} \end{array}\right]$$ (11)

### Recurrent equation-based solution

Revesz, ^17^ chose boundary conditions that need to solve the tri-diagonal system given in equation (7) where $x_{i}\;$ rational variables $e_{i}$ rational constants, *r* is a non-zero rational constant and *A* is:

$$\begin{array}{c} A=\left[\begin{array}{lllllllll} r & 1 & 0 & 0 & \cdots & 0 & 0 & 0 & 0\\ 1 & 4 & 1 & 0 & \cdots & 0 & 0 & 0 & 0\\ 0 & 1 & 4 & 1 & \cdots & 0 & 0 & 0 & 0\\ \vdots & \vdots & \vdots & \vdots & \vdots & \vdots & \vdots & \vdots & \vdots \\ 0 & 0 & 0 & 0 & \cdots & 1 & 4 & 1 & 0\\ 0 & 0 & 0 & 0 & \cdots & 0 & 1 & 4 & 1\\ 0 & 0 & 0 & 0 & \cdots & 0 & 0 & 0 & 1 \end{array}\right],\;x=\left[\begin{array}{c} \begin{array}{c} \begin{array}{c} x_{1}\\ x_{2}\\ \begin{array}{c} \vdots \\ x_{\begin{array}{c} n-1\\ x_{n} \end{array}} \end{array} \end{array} \end{array} \end{array}\right]\;\text{and}\;b=\left[\begin{array}{c} \begin{array}{l} \begin{array}{c} e_{1}\\ e_{2}\\ \begin{array}{c} \vdots \\ e_{\begin{array}{c} n-1\\ e_{n} \end{array}} \end{array} \end{array} \end{array} \end{array}\right] \end{array}$$ (12)

The first row of the new matrix in equation (12) is shown to be equivalent to the first row of the clamped *b* matrix $e_{1}$ is

$$e_{1}=\frac{3r}{2h}\left(\frac{\left(a_{1}-a_{0}\right)}{h}-f^{\prime }\;\left(x_{0}\right)\right)+\left(1\frac{r}{2}\right){\tilde{c}}_{1}$$ (13)

where, ${\tilde{c}}_{1}$ is an estimate of $c_{1}$ and *r* = 2+ √3≈3.732 . ^17^

The chosen boundary conditions are such that the first row of the new matrix was the same as that of clamped cubic spline and while that of the last row was that of the natural cubic spline fixing the value of $c_{n}$ as 0. Using equation (12), the relationships between successive spline points can be obtained as:

$$x_{i}+\frac{x_{i+1}}{r}=\sum_{0\leq k\leq \left(i-1\right)}{\left(-1\right)}^{k}\frac{e_{i-k}}{r^{k+1}}$$ (14)

Let $\propto_{0}$ , $\;\propto_{i}\;\text{for}\;1<i\;\leq \;n-1\;\text{and}\;\propto_{n}$ , respectively be:

$$\propto_{0}=0$$

$$\propto_{i}=\frac{e_{i-\propto_{i-1}}}{r}=\sum_{0\leq k\leq \left(i-1\right)}{\left(-1\right)}^{k}\frac{e_{i-k}}{r^{k+1}}$$ (15)

$$\propto_{n}=e_{n}$$

Based on the above, the closed form of solution for $x_{i}$ can be given as:

$$x_{i}=\sum_{0\leq k\leq \left(n-i\right)}{\left(\frac{-1}{r}\right)}^{-k}\propto_{i+k}$$ (16)

The above equation (16) solves $x_{i}$ no matter exactly what the initial values for $e_{i}$ . This leads to a faster evaluation of the cubic spline than solving a tri-diagonal system. The major advantage of the method is when new measurements are added to the system. While conventional tri-diagonal matrix-based algorithm requires a complete redo of the entire computation, equation (16) leads to a faster update for each *i* ≤ *n* only with the addition of the term:

$${\left(\frac{-1}{r}\right)}^{n+1-i}\propto_{n+i}$$ (17)

and $x_{n+1}=\propto_{n+1}.$ Similarly, $\propto_{i}$ constants can be updated by adding a single term $\;e_{n+1}$

The system of linear equations given in equation (7), in general, is solved by the standard solution of linear equations in the matrix form Ax=b. Alternatively, it could be solved for *n* variables by the recurrence relations given equations (16) and (17). The two methods, the first using the tri-diagonal matrix-based solution for the spline coefficients is termed the Linear Equations Method (LEM) and the second one using recurrence relations is termed the Alpha Method (AM). The algorithmic procedure for LEM and AM are given below.

### Algorithmic procedure for regular tridiagonal matrix-based Linear Equation Method (LEM)

Step 1: Given the initial vector with missing values, separate them into two sets of vectors, the observed values vector $R_{\mathrm{obs}}$ and the missing values vector $R_{\text{Miss}}$ , having sizes of *NO* and *NM*, respectively, such that *NO*+ *NM*= *N.*

Step 2: $R_{\mathrm{obs}}$ vector at $x_{i}$ values of the ( *NO*-1) splines shall be the $\;a_{i}$ coefficient vector.

Step 3: Using $\;a_{i}$ , generate the RHS vector *B* given in equation (11).

Step 4: Generate a square coefficient matrix *A* as given in equation (11)

Step 5: Solve for the $c_{i}$ vector is given in (11), using the relation *Ax* = *B*

Step 6: Applying $\;c_{i}$ in equations (8) and (9), compute the $b_{i}$ and $d_{i}$ coefficient vectors for *n*-2 points of the $R_{\mathrm{obs}}$ .

Step 7: Using the values of $\;a_{i}$ , $\;b_{i}$ , $c_{i}\;\text{and}\;d_{i}$ , missing values can be found by the equation (1) re-written as:

$${\mathrm{SF}}_{i}\left(x\right)=\left(a_{i}-b_{i}x_{i}+c_{i}x_{i}^{2}-d_{i}x_{i}^{3}\right)+\left(b_{i}-2c_{i}x_{i}+3d_{i}x_{i}^{2}\right)x+\left(c_{i}-3d_{i}x_{i}\right)x^{2}$$ (18)

Where *x* represents the missing positions, between $x_{i}$ and $x_{i+1}$ of spline *i.*

### Algorithmic procedure for recurrent equation-based Alpha Method (AM)

Step 1: Given the initial vector with missing values, separate them into two sets of vectors, the observed values vector $R_{\mathrm{obs}}$ and the missing values vector $R_{\text{Miss}}$ , having sizes of *NO* and *NM*, respectively, such that *NO*+ *NM*= *N.*

Step 2: The $R_{\mathrm{obs}}$ vector at $x_{i}$ values of the ( *NO*-1) splines is the $\;a_{i}$ coefficient vector.

Step 3: Using $\;a_{i}$ , generate the RHS vector *B* given in equation (11).

Step 4: Set $\propto_{0}=0\;\text{and}\;\propto_{n}=e_{n},$ calculate the alpha vector using the relation.

$\propto_{i}=\frac{e_{i-\propto_{i-1}}}{r}=\sum_{0\leq k\leq \left(i-1\right)}{\left(-1\right)}^{k}\frac{e_{i-k}}{r^{k+1}}$ for i values ranging from 1 to *NO*-1

Step 5: Set $x_{n}=\propto_{n}$ and solve for $c_{i}$ values using the relation.

$$c_{i}=\sum_{0\leq k\leq \left(n-i\right)}{\left(\frac{-1}{r}\right)}^{k}\propto_{i+k}$$

Step 6: Applying $\;c_{i}$ in equations (8) and (9), compute the $b_{i}$ and $\;d_{i}$ coefficient vectors for *n*-2 points of the $R_{\mathrm{obs}}$ .

Step 7: Using the values of $\;a_{i}$ , $\;b_{i}$ , $c_{i}\;\text{and}\;d_{i}$ , missing values can also be found using equation (18), re-written here again for convenience:

$${\mathrm{SF}}_{i}\left(x\right)=\left(a_{i}-b_{i}x_{i}+c_{i}x_{i}^{2}-d_{i}x_{i}^{3}\right)+\left(b_{i}-2c_{i}x_{i}+3d_{i}x_{i}^{2}\right)x+\left(c_{i}-3d_{i}x_{i}\right)x^{2}$$ (18)

Where *x* represents the missing positions, between $x_{i}$ and $x_{i+1}$ of spline *i*.

The modifications are as follows: In AM, rather than computing *E*, alpha vectors and $\;c_{i}$ coefficients for the full range of *NO*-1 data points only the RHS, *E* vector, was calculated for the full range of *NO*-1 data points, while alpha vector and $c_{i}$ were calculated only for iandi+1 data elements, where i is the missing data element. For the imputation of i the element, only the $E_{i}$ vector for all *NO*-1 data points, $\propto_{i}$ vector and $c_{i}$ vectors for iandi+1 and $b_{i}$ and $d_{i}$ coefficients were essential for the calculation $i^{\mathrm{th}}$ missing element and its imputation.

In addition, using the AM, an effective procedure was demonstrated for the computation of the following cases: (i) missing first and the last element of the data vector, (ii) missing multiple data points at the beginning and the end, and (iii) missing multiple elements anywhere in the data vector. That is in equation (18), when the current values of *A* [ *i*] are replaced either with *A* [ *N*-1] or *A* [ *i*-1] based on the position of missing edge values or continuous values the Time of Calculation (ToC) and Root Mean Squared Error (RMSE) values have improved significantly.

The formula for RMSE is:

$$\text{RMSE}=\sqrt{\frac{\sum_{i=1}^{N}{\left(\text{Predicted},-,\text{Actual}\right)}^{2}}{N}}$$

We have used RMSE and ToC as evaluation metrics to measure the effectiveness and efficiency of the proposed method because most literature used the same.

## Results and discussion

A comparison between LEM and AM is shown here for the imputation of one-min real PMU system data having a size of 1490 data points for each of the 25 heterogeneous variables obtained from five different PMUs. Since our data does not have any missing values, we artificially introduced the missing values, of 10%, 20%, 30% in random.

A sample of one-minute PMU data for five PMUs’ was used in the study. ^18^ One minute of PMU data with 10%, 20%, and 30% missing data for five PMUs were evaluated.

**Figure 1.**
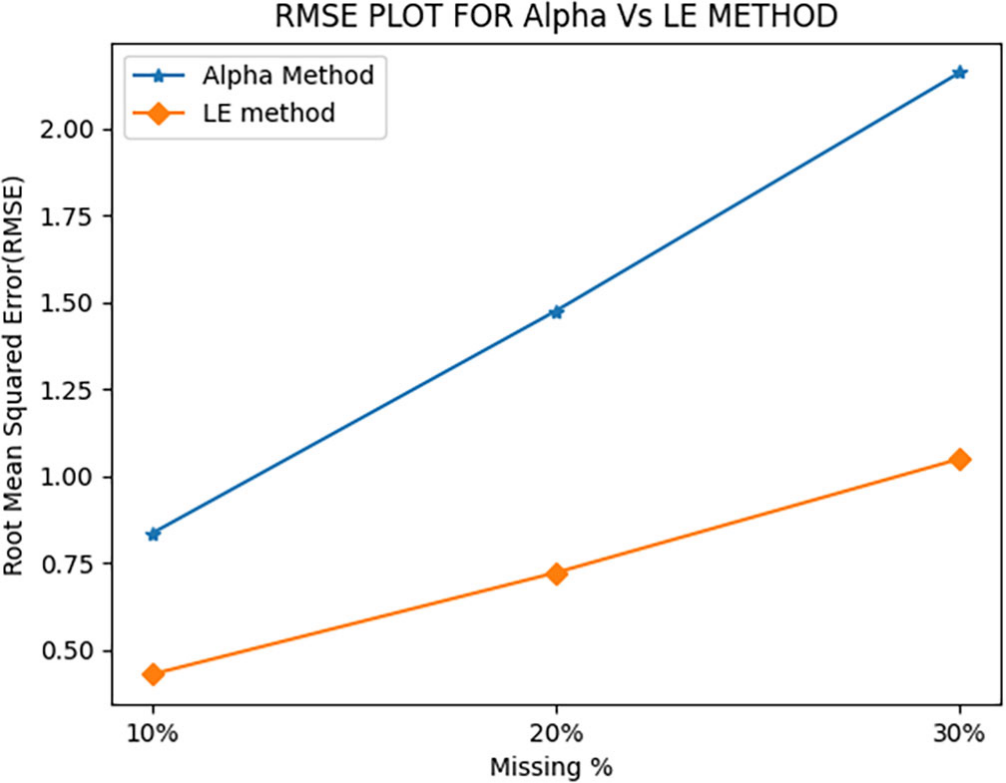
Comparison of Root Mean Squared Error (RMSE) values.

**Figure 2.**
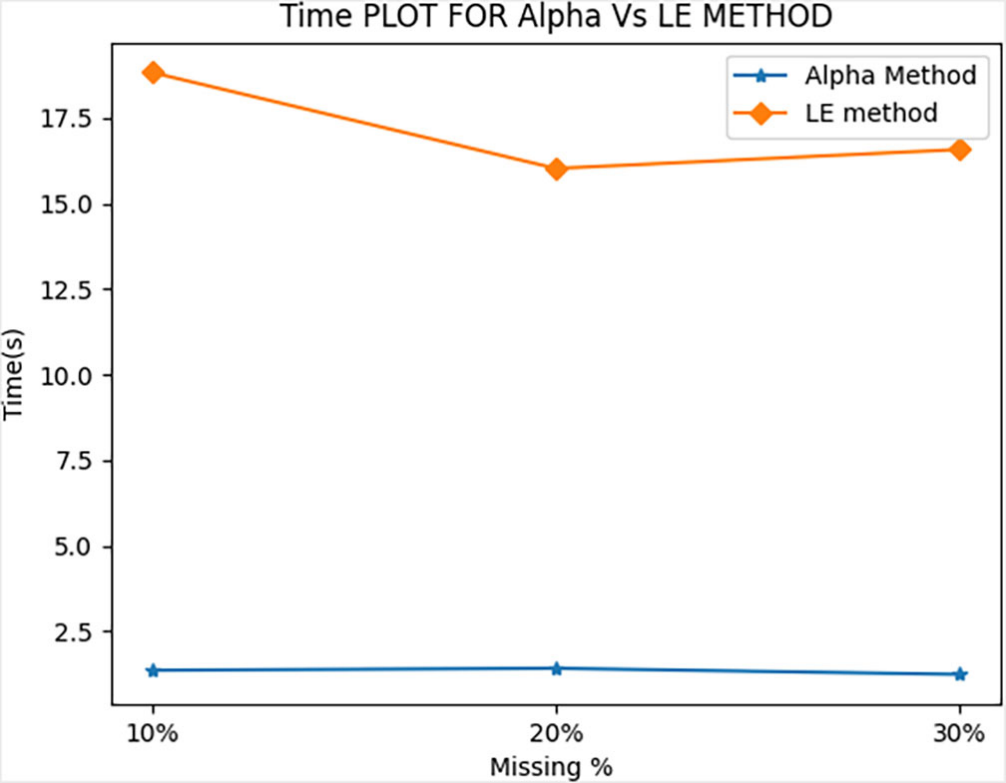
Comparison of Time of Calculation (ToC).

When AM was employed, the average RMSE values were 0.83, 1.47, and 2.16 for 10%, 20%, and 30% of missing PMU data, respectively. This can be seen in Figure 1. Moreover, for the same performance, AM showed significant improvements in its ToC as shown in Figure 2. The average ToCs for AM were 1.35, 1.41, and 1.23s when recovering 10%, 20%, and 30% of its missing data.

By comparison, LEM had ToC values of 18.83, 16.02, 16.58s for 10%, 20%, and 30% of its missing data, respectively. The proposed method reduced the ToC by a factor of approximately 10 times. LEM had higher ToC values because it needed to solve the entire set of linear equations every time it needed to find the *b _i_*, *c _i_*, and *d _i_* coefficients. On the other hand, AM only needed to calculate these coefficients at two successive points of *i* and *i*+1.

## Conclusions

In this study, AM was compared with LEM. However, because of the proliferation of the data, there is a need for customization of this technique to handle a high volume of data to reduce computational time and power. In the proposed method, the approaches demonstrated a reduced computational effort and time of calculation for solving the coefficient vectors. This study has made the following contributions: (i) the recurrent relation-based AM has been effectively employed in the imputation of PMU data and its advantages are demonstrated as an effective and efficient alternative to the conventional technique, and (ii) an effective procedure for handling missing values in special cases (edge, continuous values) is shown, which has not been addressed clearly in other methods. The proposed method has proven effective, and it only requires 10% effort in comparison to the LEM. Future research will focus on the application of the modified recurrent method in the analysis of real-time or stream PMU data.

## Data availability

### Underlying data

Harvard Dataverse: Underlying data for ‘Modified recurrent equation-based cubic spline interpolation for missing data recovery in phasor measurement unit (PMU)’, ‘PMU data’, https://doi.org/10.7910/DVN/Y2LLJJ. ^18^

This project contains the following underlying data:

- Data file: pmu1-1m-10.tab – One minute of data from PMU1 with 10% missing data
- Data file: pmu1-1m-20.tab – One minute of data from PMU1 with 20% missing data
- Data file: pmu1-1m-30.tab – One minute of data from PMU1 with 30% missing data
- Data file: pmu2-1m-10.tab – One minute of data from PMU2 with 10% missing data
- Data file: pmu2-1m-20.tab – One minute of data from PMU2 with 20% missing data
- Data file: pmu2-1m-30.tab – One minute of data from PMU2 with 30% missing data
- Data file: pmu3-1m-10.tab – One minute of data from PMU3 with 10% missing data
- Data file: pmu3-1m-20.tab – One minute of data from PMU3 with 20% missing data
- Data file: pmu3-1m-30.tab – One minute of data from PMU3 with 30% missing data
- Data file: pmu4-1m-10.tab – One minute of data from PMU4 with 10% missing data
- Data file: pmu4-1m-20.tab – One minute of data from PMU4 with 20% missing data
- Data file: pmu4-1m-30.tab – One minute of data from PMU4 with 30% missing data
- Data file: pmu5-1m-10.tab – One minute of data from PMU5 with 10% missing data
- Data file: pmu5-1m-20.tab – One minute of data from PMU5 with 20% missing data
- Data file: pmu5-1m-30.tab – One minute of data from PMU5 with 30% missing data
- README.txt

Data are available under the terms of the Creative Commons Zero “No rights reserved” data waiver (CC0 1.0 Public domain dedication).

The dataset presented in the work was obtained as real-world data from a regional Electricity authority in India. However, additional information such as the data source, the acquisition procedure, and the significance of the systemic variables are not detailed at this stage of algorithm development as the goal of this preliminary work is to demonstrate the efficacy of the proposed missing data recovery algorithm.
