# Supplementary material for: Modified recurrent equation-based cubic spline interpolation for missing data recovery in phasor measurement unit (PMU)
Source: F1000Res. 2023 Dec 18;11:246. Originally published 2022 Feb 28. [Version 3] doi: 10.12688/f1000research.73182.3 (PMC10751880; doi:10.12688/f1000research.73182.3)
Supplement: Peer review history

# Reviewer report: Reviewer response for version 3

**DOI:** 10.5256/f1000research.160163.r231067

**Published:** 2023-12-22

**Authors:** Wun She Yap

**Affiliations:** 1 Lee Kong Chian Faculty of Engineering and Science, Universiti Tunku Abdul Rahman, Kajang, Malaysia

The authors have addressed the concerns raised in my peer review report appropriately.

Is the work clearly and accurately presented and does it cite the current literature?

Partly

If applicable, is the statistical analysis and its interpretation appropriate?

Partly

Are all the source data underlying the results available to ensure full reproducibility?

Yes

Is the study design appropriate and is the work technically sound?

Yes

Are the conclusions drawn adequately supported by the results?

Partly

Are sufficient details of methods and analysis provided to allow replication by others?

Partly

Reviewer Expertise:

Artificial intelligence, cryptography, information security

I confirm that I have read this submission and believe that I have an appropriate level of expertise to confirm that it is of an acceptable scientific standard.

# Reviewer report: Reviewer response for version 2

**DOI:** 10.5256/f1000research.142368.r220241

**Published:** 2023-11-27

**Authors:** Wun She Yap

**Affiliations:** 1 Lee Kong Chian Faculty of Engineering and Science, Universiti Tunku Abdul Rahman, Kajang, Malaysia

The paper proposed a method to perform missing data recovery on PMU data. It uses a modified approach based on an established technique i.e. recurrent equation-based cubic spline interpolation. Although the paper can be difficult to follow in some sections especially the Methodology, overall, the method and its results do show some promise and is worth further investigation.

 This paper can be considered for publication if the suggestions below are addressed.

 Literature can be improved with a discussion about of some current missing data recovery techniques, especially the technique that the proposed method is based on, that is the recurrent equation-based method and tri-diagonal matrix-based conventional cubic spline interpolation. Those techniques are not discussed in the literature review despite their apparent importance to this paper.

 Although not required in this paper due to the need for conciseness and perhaps page limits, it is recommended that the authors use some visual aids/figures/plots to help explain the notion of cubic spline in future manuscripts.

 In Page 5, the section heading called "Recurrence equation-based solution" should be "Recurrent equation-based solution" for consistency with the rest of the text. Similarly, please ensure consistency throughout the paper. There are inconsistent terms such as LEM and LE (in Introduction), AM and AM Method (using method is redundant), etc.

 Please check the numbering for the equations and the references/citations to them. For example, in Page 5, the authors state "The first row of the new matrix in (6) is shown..." would imply that Equation 6 is a matrix but Equation 6 is just a normal formula.

 The dataset is lacking context but understandable due to the confidentiality of the data. It would help if more general characteristics of the dataset are given or an anonymised sampling is shown in a table. That would add depth to the discussion.

 The discussion provides valuable insights into the comparison of the two methods, but additional details and context in certain areas could enhance the clarity and completeness of the findings. For example, the use of RMSE as an evaluation metric is common in imputation tasks, but it would be helpful to know if other metrics were considered or if there are specific reasons for choosing RMSE. Similarly, why was ToC used as a metric?

 The comparison results that are provided in terms of RMSE and ToC seem to indicate that while Alpha Method has higher RMSE values compared to LE method, AM performs better in terms of time. Providing some insights in the discussion section into why this improvement occurred (e.g., the nature of the algorithm, computational complexity) would enhance the discussion.

 Can the proposed method be used for other datasets with missing data or is it optimised specially for PMU data? A brief explanation to clarify the impact of PMU data would further improve the discussion.

Is the work clearly and accurately presented and does it cite the current literature?

Partly

If applicable, is the statistical analysis and its interpretation appropriate?

Partly

Are all the source data underlying the results available to ensure full reproducibility?

Yes

Is the study design appropriate and is the work technically sound?

Yes

Are the conclusions drawn adequately supported by the results?

Partly

Are sufficient details of methods and analysis provided to allow replication by others?

Partly

Reviewer Expertise:

Artificial intelligence, cryptography, information security

I confirm that I have read this submission and believe that I have an appropriate level of expertise to confirm that it is of an acceptable scientific standard, however I have significant reservations, as outlined above.

# Reviewer report: Reviewer response for version 2

**DOI:** 10.5256/f1000research.142368.r158516

**Published:** 2023-01-03

**Authors:** Mathias Foo

**Affiliations:** 1 School of Engineering, University of Warwick, Coventry, UK

The authors have addressed all my comments and the new manuscript reads better than before.

Is the work clearly and accurately presented and does it cite the current literature?

Partly

If applicable, is the statistical analysis and its interpretation appropriate?

Partly

Are all the source data underlying the results available to ensure full reproducibility?

Partly

Is the study design appropriate and is the work technically sound?

Partly

Are the conclusions drawn adequately supported by the results?

Partly

Are sufficient details of methods and analysis provided to allow replication by others?

No

Reviewer Expertise:

Dynamical system modelling

I confirm that I have read this submission and believe that I have an appropriate level of expertise to confirm that it is of an acceptable scientific standard.

# Reviewer report: Reviewer response for version 1

**DOI:** 10.5256/f1000research.76818.r139819

**Published:** 2022-07-12

**Authors:** Shaik Mullapathi Farooq

**Affiliations:** 1 Department of Computer Science and Engineering, K. S. R. M. College of Engineering (UGC-Autonomous), Kadapa, Andhra Pradesh, India

The manuscript proposes recurrent relation based alpha method to interpolate missing PMU data. Further, the authors try to prove that the proposed method reduces computational complexity. 

 However, the comments are as follows, 

1. The Implementation of the proposed method is clearly missing (Hardware or software details used) in the manuscript which does not assist reproducing the results. 
2. Most of the manuscript is dedicated for theoretical discussion about the proposed method. But a comparison between the existing methods with the proposed method is missing. 
3. Add nomenclature that improves the readability of the manuscript. 
4. Only data set of PMU values are presented (PMU Data Harvard Data verse) instead need to add discussion about the details of PMU Data. 

Is the work clearly and accurately presented and does it cite the current literature?

Partly

If applicable, is the statistical analysis and its interpretation appropriate?

I cannot comment. A qualified statistician is required.

Are all the source data underlying the results available to ensure full reproducibility?

Partly

Is the study design appropriate and is the work technically sound?

Partly

Are the conclusions drawn adequately supported by the results?

Yes

Are sufficient details of methods and analysis provided to allow replication by others?

No

Reviewer Expertise:

Cyber security in smart grid communication network and VANET.

I confirm that I have read this submission and believe that I have an appropriate level of expertise to state that I do not consider it to be of an acceptable scientific standard, for reasons outlined above.

# Response

**Published:** 2022-12-16

**Authors:** Vik Tor Goh

**Affiliations:** Multimedia University, Malaysia

The purpose of this preliminary paper is to introduce our work in missing data recovery using cubic spline interpolation, namely the mathematical foundation and algorithmic logic. These details have been presented and explained accordingly in the paper. Additionally, the data used is also available for download by interested parties. We aim to publish a more detailed paper soon which will contain more information such as those suggested by the reviewer. Thank you for the suggestion.

 As stated earlier, the purpose of this preliminary paper is to introduce our proposed method, hence the emphasis on theoretical discussion. However, we have made an initial comparison with an existing method, namely the Linear Equation Method. This can be seen in the Results and discussion section.

 The nomenclature is improved wherever possible to improve the readability of the manuscript.

 The dataset presented in the work was obtained from a regional Electricity Authority in India. It was obtained for use as realistic data and brief details of the PMU data is now included. However, additional information such as the data source, the acquisition process, and the physical significance of the systemic variables are not detailed at this stage of algorithmic development as the main idea is only to demonstrate the efficacy of the missing data imputation algorithm. Nonetheless, we take note of this suggestion for our next submission. Thank you.

# Reviewer report: Reviewer response for version 1

**DOI:** 10.5256/f1000research.76818.r125681

**Published:** 2022-03-10

**Authors:** Mathias Foo

**Affiliations:** 1 School of Engineering, University of Warwick, Coventry, UK

In general, there is promising aspect of the proposed method but it has to be conveyed in a clearer manner. Here are my comments.

1. In Introduction section, the authors state that the comparison will be made with LEM. Can the author explain why specifically LEM is compared? Is that the current state-of-the-art method?
2. In Literature Review section, NASPI is mentioned but without proper definition of the acronym.
3. If my understanding is right, Equation (10) is a systems of linear equation of (7). Then, why does the h value in the B matrix have an exponent of 1 instead of 2 as of Equation (7)?
4. Statement above Equation (11): Unless I'm mistaken, there is no d coefficient to be solved from either equations (5) or (6).
5. Equation (11): Like Equation (10), can the authors clarify why the exponent of 1 is used for h?
6. Equation (13): Why is r taking this value? A bit more explanation would be helpful.
7. Equation (16): Why is there a 'for' in the equation?
8. Step 3 of LE method: There is no vector E in Equation (11).
9. Step 3 of AM method: There is no vector E in Equation (11).
10. Step 4 of AM method: There is no alpha term in Equation (11).
11. Results and Discussions section: Can the author explicitly write down the equation for RMSE?
  Also, I am quite surprised with the huge difference in terms of RMSE between the two methods even for the case of 10% missing data considering the same equation (18) is used for both algorithms. The difference in ToC is understandable, but the vast difference in RMSE is a bit out of my expectation. Could the author briefly comment on the plausible reason for this huge difference in the RMSE value despite both algorithm using equation (18).
12. Overall comment: The mathematical derivation is not easy to follow and there are potential mistakes in citing the equations, which makes it even harder to follow. Thus, it is difficult to ascertain whether the results can be reproduced. 

Is the work clearly and accurately presented and does it cite the current literature?

Partly

If applicable, is the statistical analysis and its interpretation appropriate?

Partly

Are all the source data underlying the results available to ensure full reproducibility?

Partly

Is the study design appropriate and is the work technically sound?

Partly

Are the conclusions drawn adequately supported by the results?

Partly

Are sufficient details of methods and analysis provided to allow replication by others?

No

Reviewer Expertise:

Dynamical system modelling

I confirm that I have read this submission and believe that I have an appropriate level of expertise to confirm that it is of an acceptable scientific standard, however I have significant reservations, as outlined above.

# Response

**Published:** 2022-12-16

**Authors:** Vik Tor Goh

**Affiliations:** Multimedia University, Malaysia

The idea of cubic spline is the development of a series of unique cubic polynomials that are fitted between the data points. Based on four continuity relations between points in the spline, the relationships between the spline coefficients shall result in a system of unique *n x n* linear equations in the matrix form *Ax = B*. The solution of this unique system of linear equations results in the values of constants at each spline point. Whenever some changes occur in any one of the splines, the system of linear equations must be solved for every specific change to fit the spline. The tri-diagonal method of solving a system of linear equations was employed in this study for comparison. Hence and from the reference, we made such a comparison to linear equation method.

 The word is already introduced in the introduction section as North American Synchro Phasor Initiative (NASPI).

 The exponent for *h* in Equation (10) and (11) should be 2 instead of 1. We have made the corrections accordingly. Thank you.

 As the reviewer correctly noted, the *d* coefficient is not solved in Equations (5) or (6). It is instead solved using Equation (9). We have made the amendments accordingly.

 The r-value in Equation (13) is a non-zero rational constant; the value is adapted from our reference work [17]. We have added this citation in the text.

 The ‘ *for*’ should not be in Equation (16). It has been corrected.

 Step 3 and Step 5 of the LE method has been corrected as vector *B* instead of *E*.

 Reference to Equation (11) in Step 4 of the AM method has been removed.

 The equation for RMSE has been added in the text.

 Upon inspection, we found that the variables *b*, *c*, and *d* of the cubic spline is found to be similar in both methods as the reviewer correctly predicted. We re-examined our results and have determined that due to an oversight, an error occurred in the final calculations of imputation values in the LE method. Instead of the coordinate numbers, the spline values at the coordinates were used for the calculation of missing values. As such, we have made the corrections to the results and discussions, as well as the plots in Figure 1 and Figure 2.

 All corrections and suggestions given were incorporated and the revised version of the paper is written.  We really appreciate the time spent by the reviewer for the useful suggestions and corrections made.
